# Brain Distribution and Modulation of Neuronal Excitability by Indicaxanthin From *Opuntia Ficus Indica* Administered at Nutritionally-Relevant Amounts

**DOI:** 10.3389/fnagi.2018.00133

**Published:** 2018-05-09

**Authors:** Giuditta Gambino, Mario Allegra, Pierangelo Sardo, Alessandro Attanzio, Luisa Tesoriere, Maria A. Livrea, Giuseppe Ferraro, Fabio Carletti

**Affiliations:** ^1^Department of Experimental Biomedicine and Clinical Neuroscience (Bio.Ne.C.), Sezione di Fisiologia Umana G. Pagano, University of Palermo, Palermo, Italy; ^2^Post-graduate School of Nutrition and Food Science, University of Palermo, Palermo, Italy; ^3^Department of Biological, Chemical and Pharmaceutical Sciences and Technologies (STEBICEF), University of Palermo, Palermo, Italy

**Keywords:** indicaxanthin, nutraceuticals, electrophysiology, microiontophoresis, excitability, neuroprotection, brain localization

## Abstract

Several studies have recently investigated the role of nutraceuticals in complex pathophysiological processes such as oxidative damages, inflammatory conditions and excitotoxicity. In this regard, the effects of nutraceuticals on basic functions of neuronal cells, such as excitability, are still poorly investigated. For this reason, the possible modulation of neuronal excitability by phytochemicals (PhC) could represent an interesting field of research given that excitotoxicity phenomena are involved in neurodegenerative alterations leading, for example, to Alzheimer’s disease. The present study was focused on indicaxanthin from *Opuntia ficus indica*, a bioactive betalain pigment, with a proven antioxidant and anti-inflammatory potential, previously found to cross blood-brain barrier (BBB) and to modulate the bioelectric activity of hippocampal neurons. On this basis, we aimed at detecting the specific brain areas where indicaxanthin localizes after oral administration at dietary-achievable amounts and highlighting eventual local effects on the excitability of single neuronal units. HPLC analysis of brain tissue 1 h after ingestion of 2 μmol/kg indicaxanthin indicated that the phytochemical accumulates in cortex, hippocampus, diencephalon, brainstem and cerebellum, but not in the striato-pallidal complex. Then, electrophysiological recordings, applying the microiontophoretic technique, were carried out with different amounts of indicaxanthin (0.34, 0.17, 0.085 ng/neuron) to assess whether indicaxanthin influenced the neuronal firing rate. The data showed that the bioelectric activity of neurons belonging to different brain areas was modulated after local injection of indicaxanthin, mainly with dose-related responses. A predominating inhibitory effect was observed, suggesting a possible novel beneficial effect of indicaxanthin in reducing cell excitability. These findings can constitute a new rationale for exploring biological mechanisms through which PhC could modulate neuronal function with a relapse on complex cognitive brain process and related neurodegenerative conditions.

## Introduction

In the recent years a growing bulk of studies expanded knowledge about the positive impact of dietary phytochemicals (PhC), or nutraceuticals, on complex physiological processes and brain aging. Many of the potential health benefits from compounds extracted by edible vegetables are based on their biological activities and the possible modulation of cellular responses. Among the numerous effects exerted by PhC, cumulative evidence has highlighted their antioxidant and anti-inflammatory properties (Ghanim et al., 2010; Obrenovich et al., 2011). Given that inflammation and oxidative stress have been reported to sustain neurological alterations leading to neurodegeneration and cognitive decline (Lassmann, 2011), the use of nutraceuticals was suggested for therapeutic approaches. In this context, specific dietary regimens including plant-derived compounds have been suggested to enhance cognitive performances and reduce neurodegenerative impairments (Spencer, 2008, 2009; Rendeiro et al., 2012; Meng et al., 2015; Almeida et al., 2016), with a remarkable significance for age-related conditions (Joseph et al., 2005; Youdim and Joseph, 2001). In the brain, excitotoxicity represents a further risk for cell survival with possible involvement in neurodegeneration (Pallo et al., 2016; Wang and Reddy, 2017). Noticeably, sound evidence highlighted the influence of PhC on cell excitability and their positive implications on cognitive functions in aging (Williams et al., 2004). Notwithstanding, only a few researches have been provided on the modulation of neuronal excitability by nutraceuticals in order to unveil new cellular targets of PhC and related beneficial employments. The present study is focused on indicaxanthin, a yellow betalain pigment contained in cactus pear fruit (*Opuntia ficus indica*). This plant, distributed in Mexico, arid regions of America, Africa, Australia and Mediterranean area (de Wit et al., 2010; Albano et al., 2015) has recently emerged as a potential source of valuable components for human health and food industry (Feugang et al., 2006; Kim et al., 2012; Heinrich et al., 2014; Sánchez et al., 2014; Alencar et al., 2018). Indicaxanthin shares many of the favorable effects of PhC, as described in previous scientific reports (Butera et al., 2002). Indeed, indicaxanthin has proven its antioxidant potential both by preventing lipid peroxidation and reducing reactive oxygen/nitrogen species (Allegra et al., 2005, 2007; Tesoriere et al., 2006), but also by interfering with specific redox-dependent signaling pathways in experimental models of innate immunity *in vitro* (Allegra et al., 2014a; Tesoriere et al., 2015). Relevantly and in line with its redox-modulating effects, the pigment has also been shown to exert significant anti-inflammatory effects *in vivo* (Allegra et al., 2014b). All the effects exerted by indicaxanthin gain a more interesting value in the light of its remarkable bioavailability *in vivo* (Tesoriere et al., 2004). More recently a multidisciplinary approach has demonstrated that indicaxanthin orally administered at dietary-relevant doses is able to cross blood-brain barrier (BBB) and accumulate within the rat brain; beyond this, the intriguing ability of this phytochemical to modulate the bioelectric activity of hippocampal neurons for the first time has emerged (Allegra et al., 2015). In this light, the current research aimed to detect a brain distribution pattern of indicaxanthin, after oral administration of nutritionally-relevant amounts, by HPLC assay. Afterwards, microiontophoretic recordings were carried out to explore a possible influence of the indicaxanthin on the bioelectric activity of single neuronal units in specific brain areas.

Therefore, the present study is addressed to shed new light on the direct modulation of membrane excitability by edible PhC. In particular, this could unveil the nutritional importance of indicaxanthin and its influence on a pivotal aspect of neuron physiology, with a possible impact on the conditions of altered cell excitability that often underlie neuronal damages and, ultimately, cognitive dysfunctions.

## Materials and Methods

### Animals

Adult male Wistar rats (Morini, Milan, Italy) weighing 220−280 g were used in all experiments. Animals had access to food and water *ad libitum*. The light cycle was automatically controlled (on at 07:00, off at 19:00) and the room temperature was thermostatically regulated at 22° ± 1°C.

All procedures were performed in strict accordance with the European directive 2010/63/EU and the institutional guidelines, authorized by the Italian Ministry of Health (authorization no. 258-95-A) and approved by the Committee for the Protection and Use of Animals of the University of Palermo. All efforts were made to minimize animal suffering and to reduce the number of animals used.

### Quantitative Assessment of Indicaxanthin Concentration in Selected Brain Areas

#### Reagents

Unless stated otherwise, all reagents were from Sigma-Aldrich (Milan, Italy) and of the highest grade commercially available.

#### Extraction and Purification of Indicaxanthin From Cactus Pear Fruits

Indicaxanthin was isolated from cactus pear (*Opuntia ficus-indica*) fruits (yellow cultivar). Briefly, the phytochemical was separated from a methanol extract of the pulp by liquid chromatography on Sephadex G-25 as previously reported (Allegra et al., 2015). Fractions containing the pigment were submitted to cryodesiccation, purified as described (Allegra et al., 2015), quantified by HPLC as below reported, and suspended in PBS for the experiments.

#### Surgical Procedure

Rats (*n* = 4) received intragastric administration of indicaxanthin (2 μmol/kg) or saline 0.9%. According to previous pharmacokinetic study of indicaxanthin maximal brain levels, animals were sacrificed after 1 h with pentobarbital and perfused with normal saline to remove any compounds still circulating in the blood, then brains were removed. Cerebral sections, including cortex, hippocampus, striato-pallidal complex, diencephalon (thalamic and subthalamic regions), brainstem and cerebellum, were dissected and harvested following standardized coordinates for chromatographic quantification as previously reported (Paxinos and Watson, 1998; Chan et al., 2010; Zhang et al., 2015). In particular, discrete brain tissues were maintained on a dry ice bed, weighed, wrapped in aluminum foil to protect against light and stored at −20°C until sample analysis.

#### Quantification of Indicaxanthin in Brain Samples

The amount of indicaxanthin within brain samples was evaluated as reported below. With regard to each cerebral section, tissues were carefully washed with saline, pooled and homogenized in PBS. Indicaxanthin was extracted from samples with chloroform: methanol, 2:1 by volume (1 g of tissue with 3 volumes of extraction mixture). The methanol phase from all samples at each time point was dried under nitrogen, re-suspended in 1% acetic acid in water, analyzed on a Varian Microsorb C-18 column (4.6 Å ~250 mm; Varian, Palo Alto, CA, USA), and eluted with a 20-min linear gradient elution from solvent A (1% acetic acid in water) to 20% solvent B (1% acetic acid in acetonitrile) with a flow rate of 1.5 mL/min. Spectrophotometric revelation was at 482 nm. Under these conditions, indicaxanthin eluted after 8.15 min and was quantified by reference to standard curves constructed with 0.2−10 ng of purified compounds and by relating its amount to the peak area.

### Electrophysiological Assessment of Indicaxanthin Effect in Different Brain Areas

#### Surgical Procedures

Adult male Wistar rats (*n* = 8) were anesthetized with urethane (Sigma Chemical Co., St. Louis, MO, USA) at the dose of 1.2 g/kg i.p. and positioned in a stereotaxic apparatus (David Kopf Instruments, Tujunga, CA, USA). Body temperature was maintained at 37–38°C using a heating pad. The skull was exposed and holes were drilled. Coordinates were determined using a stereotaxic atlas, anterior (A) from interaural line, lateral (L) from midline, ventral (V) to cortical surface (Paxinos and Watson, 1998): cortex (A: 4.7–10.4, L: 1–5, V: 1–2.5), hippocampus (A: 4.8–5.8, L: 1–4, V: 2–3.5), striatum (A: 7.4–10.7, L: 1.5–5, H: 3–8), globus pallidus (A: 7–8.2, L: 2.6–4, H: 5–7), thalamus (A: 5.2–7.4, L: 2–4, V: 4–6.5) and subthalamic nucleus (A: 4.7–5.4, L: 2–3.2, H: 6.8–8).

#### Electrophysiological Recordings

**Figure 1:**
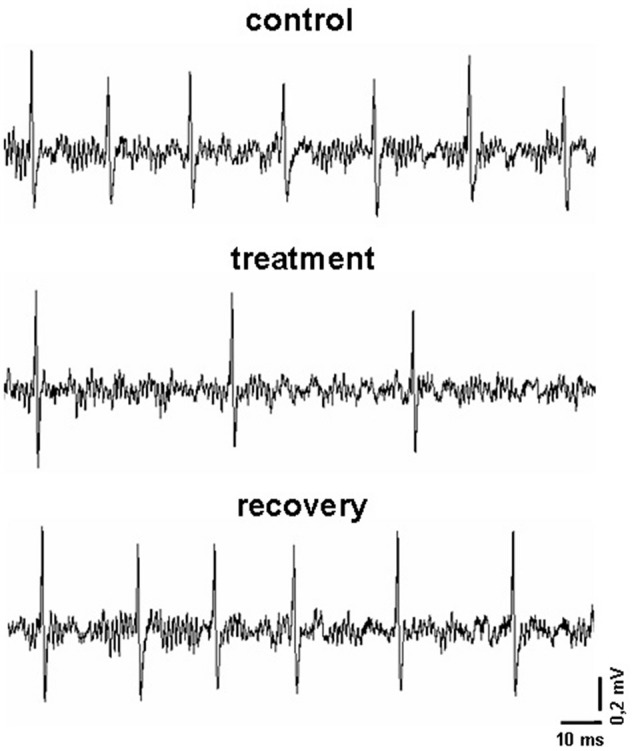
Representative recordings obtained in an experimental session. In particular, it is displayed the firing pattern of a pallidal neuron before, during and after the microiontophoretic treatment with indicaxanthin (0.17 ng/neuron).

A seven-barrel glass micro-electrode was used for both recording and drug ejection, as previously reported (Carletti et al., 2009). The center recording barrel (1.1−2.0 MΩ resistance) was filled with 2 M NaCl with 1% Fast Green (Sigma), one side barrel was filled with 2 M NaCl 0.9% buffered saline solution (for automatic current balancing), and the others were filled respectively with 12 μM, 6 μM and 3 μM indicaxanthin at pH 7.4, prepared by dilution 1:1000 of stock solution of indicaxanthin 12 mM, 6 mM and 3 mM, respectively. Negative currents of 8−10 nA were applied to the barrels (20−70 MΩ) to retain drugs (Neurophore BH-2 System, Harvard Apparatus, Hamden, CT, USA). To concentrate the drug at the pipette tip, at the beginning of each track, maximal ejection currents were applied to each barrel for 30 min. Under these conditions a spontaneous discharge was evident for all recorded cells (Figure 1). Amplification, filtering and acquisition of recordings was performed for off-line analysis as previously described (Carletti et al., 2016). Neuronal activity was continuously displayed and updated online every 5 s via a rate-meter histogram with a counter window on the computer screen to detect any variations of neuronal firing rate. All computer operations were performed using the SciWorks package, version 5.0 (Datawave Technologies, Loveland, CO, USA). Neuronal units were not taken into consideration if marked changes in amplitude or configuration of the spike were observed, or if there was early death of the cell in the course of recording. Baseline activity of each neuron was recorded for 3−5 min before acute vehicle or drug administration (Figure 1).

#### Indicaxanthin Treatment

As previously described (Allegra et al., 2015), pilot recordings with the maximal concentration of 12 μM indicaxanthin (below reported) were carried out at increasing ejection currents to identify the minimal current exerting an effect on all responsive neurons in each brain area of investigation. For this purpose, neurons were tested with 90 s pulses of indicaxanthin at 40, 60 and 80 nA ejection currents, with a 90 s interpulse interval to prevent any influence of the previous pulse on the following one. The minimal current effective on all responsive neurons resulted to be 60 nA. After this step, all recorded neurons underwent the iontophoretic administration of indicaxanthin at different amounts for 5 min with ejection current of 60 nA to correlate eventual modifications of neuronal response with the amount of the drug. Once the application of indicaxanthin was terminated, recording continued until the spontaneous firing rate recovered to baseline (Figure 1). Control tests, applying the same ejection currents used for the treatments, were performed with vehicle solutions and no influence on the neuronal firing were found. It is worth to highlight that ejection times and currents, in our microiontophoretic approach, result in a total amount of indicaxanthin interacting with single neurons in a nanogram range. Indeed, indicaxanthin released from a barrel in 5 min at 60 nA was equivalent to 0.1 μl, corresponding approximatively to 0.34 ng (for 12 μM), 0.17 ng (for 6 μM), and 0.085 ng (for 3 μM). Apart from brain localization, microiontophoretic recordings were carried out in telencepahalic and diencephalic regions (cortex, hippocampus, striatum, globus pallidus, thalamus and subthalamic nucleus), involved in cognitive processes and mostly investigated by our group in previous electrophysiological researches (Sardo et al., 2003, 2006, 2009, 2012; Carletti et al., 2012, 2017; Plescia et al., 2014). Hindbrain areas were not taken in consideration for recordings because of stereotaxic limitations. Lastly, at the end of each experiment, the recording site was marked with Fast Green through the electrode, using a 50 mA ejection current for 15 min. After transcardial perfusion with saline followed by 10% buffered formalin, brains were removed and cryoprotected in 30% sucrose/PBS. Coronal frozen sections were cut at 50 mm and stained with cresyl violet for histological verification and reconstruction of recording sites (Supplementary Figure S1). Images of sections were acquired using a Leica DFC camera attached to a stereomicroscope (Leica Microsystems Imaging solutions Ltd., Cambridge, UK).

#### Microiontophoretic Data Analysis

Neuronal firing rate was off-line analyzed before, during and after drug administration for each recorded unit. Individual rate-meter histograms (5 s bin width) were analyzed by means of a nonparametric Mann−Whitney *U* test to detect any statistically significant treatment-related change in neuronal firing. To analyze the effects induced by treatments, neurons were considered responsive if changes were significant (probability level *P* < 0.05) for at least six consecutive bins for indicaxanthin, the first of which was respectively labeled as the onset of a response. The intensity of the effect on neuronal firing (changes in the discharge with respect to baseline, considered as 1 min recording before treatment) was expressed as percentage magnitude (hereafter, magnitude), rather than absolute values, in order to normalize the responses of neurons with different baseline discharge frequencies. The latency of the effect was considered as the time from the appearance of the effect, and the duration as its timespan. Comparisons of the effects of different amounts of indicaxanthin on the mean magnitude of discharge frequencies, latency and duration were performed by means of a Mann–Whitney *U* test. For all statistical tests used, the null hypothesis was rejected at a *P* lower than 0.05. If not otherwise indicated, all results are expressed as mean ± SD.

## Results

### Indicaxanthin Selectively Distributes Within Brain Areas

**Figure 2:**
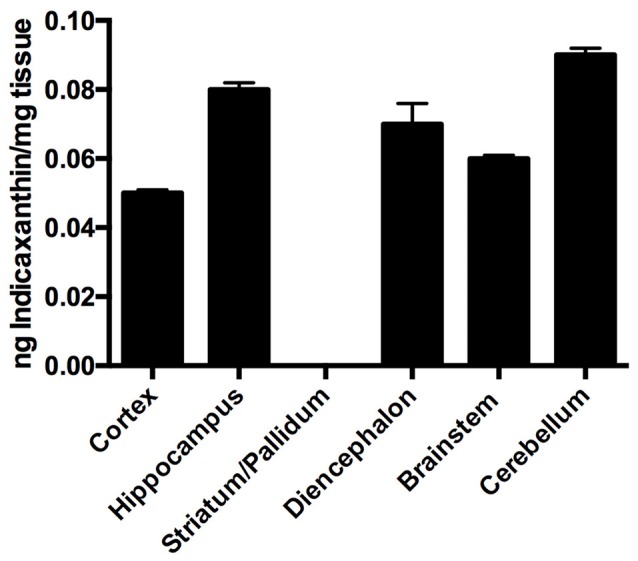
Distribution of indicaxanthin in selected rat brain areas 60 min after ingestion of 2 μmol/kg, expressed as ng/mg tissue. Values are indicated as means ± SEM.

Distribution of indicaxanthin in rat brain (*n* = 4) was investigated by assessing the amount of the pigment accumulated in selected brain areas (i.e., cortex, hippocampus, striato-pallidal complex, diencephalon, cerebellum and brainstem) per mg of fresh tissue, 1 h after oral administration of 2 μmol/Kg. Interestingly, indicaxanthin accumulated in all examined areas with the exception of striato-pallidal complex (Figure 2) and no known metabolite was recovered in brain samples (not shown). In particular, indicaxanthin reached a maximum and minimum amount of the pigment in cerebellum and cortex (0.09 ± 0.002) and (0.05 ± 0.002) ng/mg of fresh tissue, respectively (Figure 2).

### Microiontophoretic Recordings

#### Cortex

**Figure 3:**
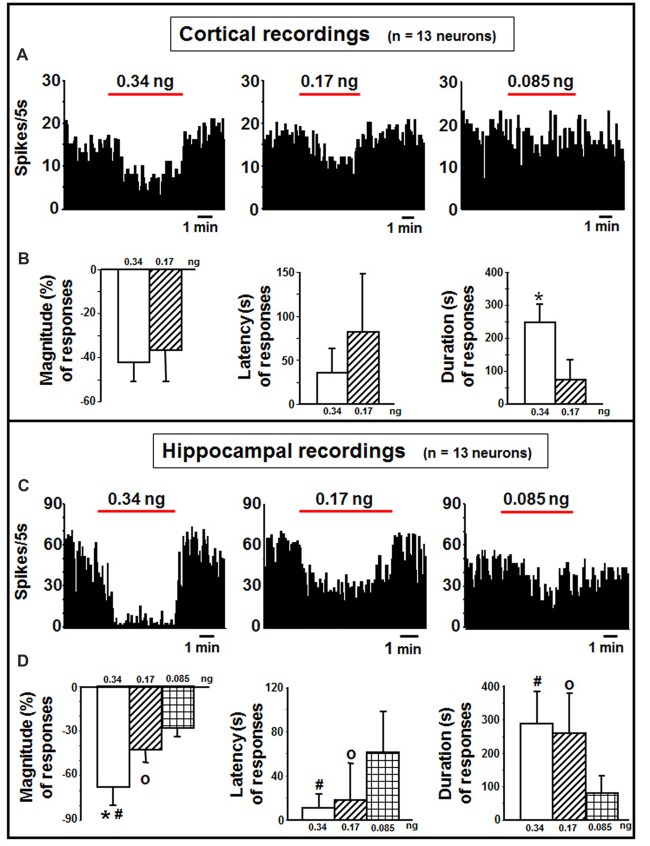
Cortical and hippocampal recordings. **(A)** Representative rate-meter histogram (bin width 5 s) showing the firing rate of a cortical neuron following indicaxanthin administration at various amounts (0.34, 0.17, 0.085 ng/neuron) for 5 min. **(B)** Bar histogram for magnitude, latency and duration showing the significant effect of indicaxanthin at different amounts (0.34, 0.17 ng/neuron) on cortical neuronal responses (*n* = 13). Each bar represents mean values (± standard deviation; *statistically significant effect of indicaxanthin 0.34 vs. 0.17 ng/neuron). (Statistical significance for *P* < 0.05). **(C)** Representative rate-meter histogram (bin width 5 s) showing the firing rate of a hippocampal neuron following indicaxanthin administration at various amounts (0.34, 0.17, 0.085 ng/neuron) for 5 min. **(D)** Bar histogram for magnitude, latency and duration showing the significant effect of indicaxanthin at different amounts (0.34, 0.17, 0.085 ng/neuron on hippocampal neuronal responses (*n* = 13). Each bar represents mean values (± standard deviation; *statistically significant effect of indicaxanthin 0.34 vs. 0.17 ng/neuron; ^#^statistically significant effect of indicaxanthin 0.34 vs. 0.085 ng/neuron; °statistically significant effect of 0.17 vs. 0.085 ng/neuron). Statistical significance for *P* < 0.05.

Cortical neurons (*n* = 13 cells) were administered with indicaxanthin ejected at 60 nA for 5 min at 0.34, 0.17 and 0.085 ng/neuron (Figure 3A). Significant inhibition of firing rate was recorded after indicaxanthin administration at 0.34 ng/neuron in 9 cells (69.23%, *P* < 0.05) with a mean magnitude of −41.89 ± 9.59%, a mean latency of 28.89 ± 23.29 s and a mean duration of 246.56 ± 58.41 s (Figure 3B). Administration of indicaxanthin at 0.17 ng/neuron reduced neuronal discharge in 7 cells (53.85%, *P* < 0.05) showing a mean magnitude of −36.38 ± 15.06%, a mean latency of 66.43 ± 79.83 s and a mean duration of 75.00 ± 75.17 s (Figure 3B). No effects were observed for treatment with indicaxanthin at 0.085 ng/neuron. Between-treatment analysis showed that only the duration of the effect was significantly higher for indicaxanthin at 0.34 with respect to 0.17 ng/neuron (*Z* = −3.07, *P* = 0.0021), while magnitude and latency were not modified between different amounts of the pigment (Figure 3B).

#### Hippocampus

Neurons belonging to the hippocampus (*n* = 13 cells) were treated with indicaxanthin ejected at 60 nA for 5 min at 0.34, 0.17 and 0.085 ng/neuron (Figure 3C). Indicaxanthin at 0.34 ng/neuron induced significant inhibition in firing rate in 10 cells (76.92%, *P* < 0.05) with a mean magnitude of −67.85 ± 12.57%, a mean latency of 12.00 ± 13.17 s and a mean duration of 287.50 ± 98.86 s (Figure 3D). Administration of 0.17 ng/neuron indicaxanthin at 60 nA caused inhibition of neuronal discharge in 8 cells (61.54%, *P* < 0.05) showing a mean magnitude of −42.82 ± 8.85%, a mean latency of 20.63 ± 32.78 s and a mean duration of 261.25 ± 118.98 s (Figure 3D). Lastly, indicaxanthin at 0.085 ng/neuron reduced firing rate of 6 neurons (46.15%, *P* < 0.05) revealing a mean magnitude of −27.78 ± 7.82%, a mean latency of 60.83 ± 38.39 s and a mean duration of 79.17 ± 54.90 s (Figure 3D). Between-treatment comparisons revealed that the effect of indicaxanthin was markedly dose-dependent. In detail, considering magnitude values, the inhibition by indicaxanthin at 0.34 was higher than the effect of indicaxanthin at both 0.17 (*Z* = −3.199, *P* = 0.0014) and 0.085 ng/neuron (*Z* = −3.254, *P* = 0.0011), and a significant reduction was observed comparing effect at 0.17 with that at 0.085 ng/neuron (*Z* = −2.453, *P* = 0.0142). The latency of the effect was reduced comparing 0.34–0.085 ng/neuron (*Z* = −2.82, *P* = 0.0048) and 0.17–0.085 ng/neuron (*Z* = −2.19, *P* = 0.0282). Lastly, with regard to the duration of the effect, the inhibition was enhanced at 0.34 and at 0.17, respectively, vs. 0.085 ng/neuron (*Z* = −3.037, *P* = 0.0024 and *Z* = −2.84, *P* = 0.0045; Figure 3D).

#### Striatum

**Figure 4:**
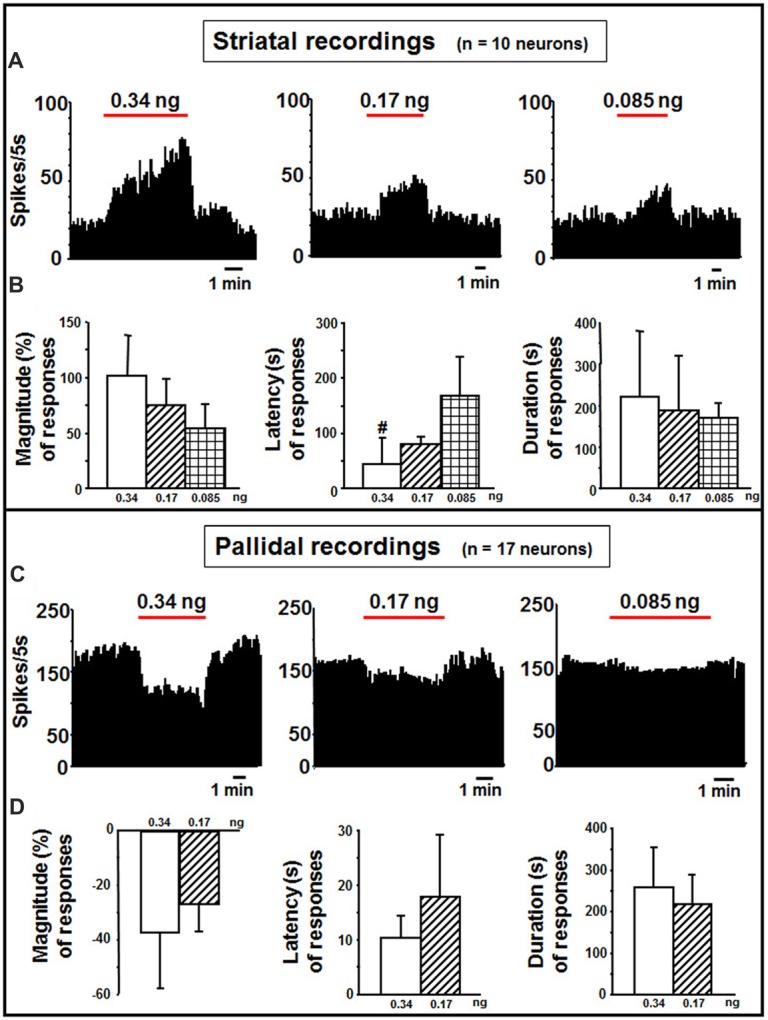
Striatal and pallidal recordings. **(A)** Representative rate-meter histogram (bin width 5 s) showing the firing rate of a striatal neuron following indicaxanthin administration at various amounts (0.34, 0.17, 0.085 ng/neuron) for 5 min. **(B)** Bar histogram for magnitude, latency and duration showing the significant effect of indicaxanthin at different amounts (0.34, 0.17, 0.085 ng/neuron) on striatal neuronal responses (*n* = 10). Each bar represents mean values (± standard deviation; ^#^statistically significant effect of indicaxanthin 0.34 vs. 0.084 ng/neuron). Statistical significance for *P* < 0.05. **(C)** Representative rate-meter histogram (bin width 5 s) showing the firing rate of a pallidal neuron following indicaxanthin administration at various amounts (0.34, 0.17, 0.085 ng/neuron) for 5 min. **(D)** Bar histogram for magnitude, latency and duration showing the significant effect of indicaxanthin at different amounts (0.34, 0.17, 0.085 ng/neuron) on pallidal neuronal responses (*n* = 17). Each bar represents mean values (± standard deviation). Statistical significance for *P* < 0.05.

In the striatum, indicaxanthin at 0.34, 0.17 and 0.085 ng/neuron was injected at 60 nA for 5 min to 10 neurons (Figure 4A). The highest amount caused excitation in 5 cells (50%, *P* < 0.05), whilst the other amounts in 3 cells (30%, *P* < 0.05). The effect of indicaxanthin at 0.34 ng/neuron presented a mean magnitude of 104.62 ± 32.30%, a latency of 44.00 ± 45.88 s and a duration of 223.00 ± 159.24 s (Figure 4B). Excitation by indicaxanthin at 0.17 ng/neuron showed a mean magnitude of 75.74 ± 18.02%, a latency of 80.00 ± 39.05 s, and a duration of 188.33 ± 136.50 s (Figure 4B). Lastly, indicaxanthin at 0.085 ng/neuron produced an excitation characterized by a mean magnitude of 53.43 ± 24.34%, a latency of 166.67 ± 70.06 s, and a duration of 170.00 ± 39.69 s (Figure 4B). Between-treatment analysis on the different concentrations of indicaxanthin revealed that the latency of the effect was significantly shorter at 0.34 vs. at 0.085 ng/neuron (*Z* = −2.23, *P* = 0.0253; Figure 4B).

#### Globus Pallidus

Pallidal neurons (*n* = 17 cells) was administered with indicaxanthin with current of 60 nA for 5 min at 0.34, 0.17 and 0.085 ng/neuron (Figure 4C). In 15 cells (88.24%, *P* < 0.05) a reduction of firing was observed after administration of indicaxanthin at 0.34 ng/neuron. Mean magnitude of firing was of −36.81 ± 20.94%, mean latency 10.33 ± 3.99 s and mean duration 258.33 ± 95.71 s (Figure 4D). Inhibition of neuronal discharge was found in 9 neurons (52, 94%, *P* < 0.05), treated with indicaxanthin at 0.17 ng/neuron with a mean magnitude of −26.55 ± 10.38%, mean latency 17.78 ± 11.49 s and mean duration 217.22 ± 67.37 s (Figure 4D). No effects were observed with indicaxanthin at 0.085 ng/neuron. Statistical analysis between concentrations revealed that discharge parameters were not modified comparing 0.34 with 0.17 ng/neuron.

#### Thalamus

**Figure 5:**
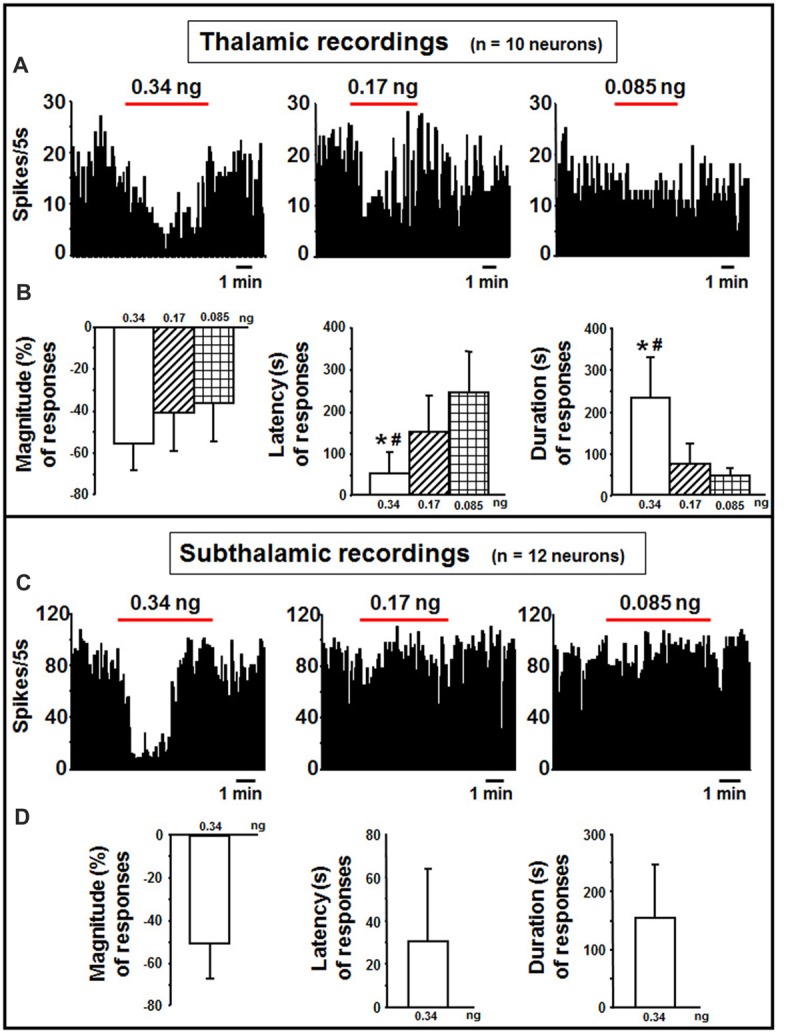
Thalamic and subthalamic recordings. **(A)** Representative rate-meter histogram (bin width 5 s) showing the firing rate of a thalamic neuron following indicaxanthin administration at various amounts (0.34, 0.17, 0.085 ng/neuron) for 5 min. **(B)** Bar histogram for magnitude, latency and duration showing the significant effect of indicaxanthin at different amounts (0.34, 0.17, 0.085 ng/neuron) on thalamic neuronal responses (*n* = 10). Each bar represents mean values (± standard deviation; *statistically significant effect of indicaxanthin 0.34 vs. 0.17 ng/neuron; ^#^statistically significant effect of indicaxanthin 0.34 vs. 0.085 ng/neuron). Statistical significance for *P* < 0.05. **(C)** Representative rate-meter histogram (bin width 5 s) showing the firing rate of a subthalamic neuron following indicaxanthin administration at various amounts (0.34, 0.17, 0.085 ng/neuron) for 5 min. **(D)** Bar histogram for magnitude, latency and duration showing the significant effect of indicaxanthin at 0.34 ng/neuron on subthalamic neuronal responses (*n* = 12). Each bar represents mean values (± standard deviation). Statistical significance for *P* < 0.05.

In the thalamus, indicaxanthin was administered at 0.34, 0.17 and 0.085 ng/neuron at 60 nA current for 5 min in 10 neurons (Figure 5A). After indicaxanthin administration at 0.34 ng/neuron, 9 neurons (90%, *P* < 0.05) exhibited inhibition of firing rate with a mean magnitude of −55.46 ± 12.58%, a mean latency of 56.11 ± 61.84 s and a mean duration of 231.67 ± 97.60 s (Figure 5B). Furthermore, administration of indicaxanthin at 0.17 ng/neuron induced reduction of neuronal discharge in 5 cells (50%, *P* < 0.05) with a mean magnitude of −38.99 ± 19.83%, a mean latency of 151.00 ± 85.32 s and a mean duration of 74.00 ± 50.17 s (Figure 5B). Lastly, the administration of indicaxanthin at 0.085 ng/neuron reduced discharge in 3 neurons (30%, *P* < 0.05) with a mean magnitude of −35.75 ± 18.40%, latency of 243.33 ± 97.51 s and duration of 45.00 ± 25.98 s (Figure 5B). As for between-treatment analysis, the latency of the effect was reduced at 0.34 vs. 0.17 (*Z* = −2.067, *P* = 0.0388) and 3 mM (*Z* = −2.126, *P* = 0.0335). Also, the duration was enhanced at 0.34 compared to 0.17 (*Z* = −2.733, *P* = 0.0063) and to 0.085 ng/neuron (*Z* = −2.496, *P* = 0.0126; Figure 5B).

#### Subthalamic Nucleus

Subthalamic neurons (*n* = 12) were treated with indicaxanthin at 0.34, 0.17 and 0.05 ng/neuron at 60 nA currents for 5 min (Figure 5C). Eleven of them were inhibited by the highest concentration (91.6%, *P* < 0.05). The effect showed a mean magnitude of −50.15 ± 16.52%, a latency of 33.45 ± 33.43 s, and a duration of 154.55 ± 92.10 s (Figure 5D). The administration of lower amounts did not influence discharge activity.

## Discussion

Biodistribution and neurophysiological data from this study fall within the remit of intense research on PhC and human health, focusing on the interaction of indicaxanthin with central nervous system. Within the immense pharmacological cornucopia of nutraceuticals, only few of them (curcumin, resveratrol, tea polyphenols) have been reported to cross the BBB and accumulate in different brain areas exerting neuroprotective effects (Milbury and Kalt, 2010; Vingtdeux et al., 2010; Pandareesh et al., 2015). In the present research, we demonstrate for the first time that indicaxanthin, a betalain pigment able to cross the BBB (Allegra et al., 2015), when administered at nutritionally-relevant amounts accumulates within different brain areas and remains unmodified at levels comparable or even greater than those showed by other PhC (Vingtdeux et al., 2010; Vanmierlo et al., 2012). Moreover, while some PhC distribute within the brain homogeneously (Milbury and Kalt, 2010), we here show that indicaxanthin gains a specific access to selected brain areas. This phytochemical was indeed found in different amount in cortex, hippocampus, diencephalon, brainstem, cerebellum, but not in the striato-pallidal complex. While indicaxanthin peculiar ability to cross BBB may be due to its amphiphilicity, high bioavailability and affinity to lipid membranes (Turco Liveri et al., 2009), a more complex scenario maybe envisaged to explain the selective accumulation of the pigment in some brain areas and the exclusion from others such as the striato-pallidal complex. Anatomically speaking, the discrepancy could be ascribed to the peculiar structural features of this subcortical region belonging to the basal ganglia; in particular, the presence of different fiber bundles, encapsulating the striatum and pallidum, could not allow indicaxanthin accumulation as easily as in the rest of the brain (Parent and Hazrati, 1995). The chemical structure of indicaxanthin could also account for its absence in some brain areas unlike, for example, the anthocyanins contained in the blueberry, capable to reach the striatum after oral consumption (Andres-Lacueva et al., 2005). Surely enough, concentrations of indicaxanthin necessary to enter in distinct brain structures can vary on the basis of the mechanism employed for the passage, for instance membrane transporters or endocytosis, and this could also be responsible for the lack of indicaxanthin accumulation both in the striatum and pallidum. This aspect surely deserves further studies focusing on the interaction of indicaxanthin with the different biological barriers lying in the brain.

Considering the permeability of indicaxanthin to BBB and its distribution, we extensively investigated the neuromodulatory potential of indicaxanthin. To this purpose, electrophysiological recordings were conducted in the brain areas mainly involved in forebrain cognitive functions, including striato-pallidal complex; single neuronal unit recordings associated with microiontophoresis represent an adequate and direct method to examine the electrophysiological outcomes of exogenous substances with unknown properties. Furthermore, administrations of indicaxanthin at three different amounts were carried out to trace a dose-response profile (Table 1). In detail, during cortical recordings, the evaluation of cell firing revealed that discharge frequency was inhibited by indicaxanthin at 0.34 and 0.17, but not at 0.085 ng/neuron. The inhibition induced by indicaxanthin at 0.34 appeared more intense than that at 0.17/ng neuron and characterized by an increased magnitude of effect accompanied by a lower latency and a greater duration of the effect, though statistical significance between effects at different amount of the molecule was observed only for the effect duration. This ability of indicaxanthin to modulate neuronal excitability emerges more clearly in the hippocampus, where the phytochemical always resulted effective. Indeed, the different amounts of indicaxanthin administered induced a dose-dependent significant inhibition of cell firing, especially on the magnitude of the effect. This was confirmed by the related changes in latency and duration of the effect. These data suggest that hippocampal neurons are more responsive to indicaxanthin with respect to cortical ones. In the striatum, we observed an opposite outcome since indicaxanthin determined the excitation of firing rate with an intensity related to the amount applied. In particular, the greater magnitude of the effect was associated to shorter latency and longer duration of the response, although statistical significance came out only for the latency between 0.34 vs. 0.085 ng/neuron. In the globus pallidus, belonging to basal ganglia circuit as well as striatum, indicaxanthin has instead induced the inhibition of neuronal firing, but only after the injection at 0.34 vs. 0.085 ng/neuron, that did not statistically differ between them. As for recordings performed in the thalamus, it was evidenced a marked reduction of neuronal excitability. Amount-related changes in magnitude were found and were confirmed by significant differences between doses in latency and duration. Finally, the subthalamic nucleus was pointed out as the areas that was less responsive to treatments since indicaxanthin was effective only at 0.34 ng/neuron in reducing cell firing.

**Table 1:** Effects on neuronal firing rate in different brain structures after microiontophoretic administration of three amounts of indicaxanthin.

	0.34 ng	0.17 ng	0.085 ng
Cortex	−	−	°
Hippocampus	−	−	−
Striatum	+	+	+
Globus pallidus	−	−	°
Thalamus	−	−	−
Subthalamic Nucleus	−	°	°

*Amounts (ng) refer to the indicaxanthin ejected per neuron by microinthophoresis in 5 min at 60 nA. Neurons belonging to each structure displayed either excitatory or inhibitory effects. Excitatory, inhibitory and null responses are indicated with (+), (−), (°), respectively*.

This evidence sustains a prevalent inhibitory effect of indicaxanthin on discharge activity of neurons in rat brain (Table 1). As for the exclusive excitatory effect observed in the striatum, in order to explain this different result, it should be noted that all the other structures examined are mainly based on glutamatergic or GABAergic transmission, while in the striatum a relevant dopaminergic influence integrate glutamatergic input and contribute to the neuronal excitability (Parent and Hazrati, 1995; Michaelis, 1998). Therefore, the modulatory action of indicaxanthin occurred in a peculiar synaptic environment, more finely regulated, possibly underlying the increase in discharge frequency. Interestingly, in our previous researches the striatum already showed its particularity, given that the microiontophoretic administration of nitric oxide donors, likely intervening in glutamatergic and GABAergic transmission, induced excitation in all basal ganglia structures, except for the striatum where inhibition was observed (Sardo et al., 2003, 2006, 2009, 2011; Carletti et al., 2009, 2012).

Taking into account the areas inhibited, indicaxanthin action could be framed within the context of neurotransmission systems exploiting glutamate and GABA, as already mentioned. In this view, the inhibition of neuronal activity could be due to the reduction of glutamate neurotransmission or the enhancement of GABA transmission. Although our data do not allow to assert the presence of a direct interaction with these systems, our previous article demonstrated the potential affinity of indicaxanthin to subunit N2A of NMDA glutamate receptor (NMDAR), that is pivotal for NMDAR function but is also involved in alteration linked to aging neurodegeneration (Gardoni et al., 2006; Allegra et al., 2015). Moreover, the ability of indicaxanthin to reduce glutamate-mediated neuronal excitability in the hippocampus was previously outlined (Allegra et al., 2015). In support of this interpretation, other researches propose the interaction between PhC and glutamate receptors and suggest, for example, that a diet enriched with blueberry ameliorated hippocampal learning process by preventing dysfunctions in the glutamatergic transmission (Williams et al., 2004; Coultrap et al., 2008). High expression of NMDAR subunit N2A in both the cortex and hippocampus suggests that this could be a potential site of action for indicaxanthin in these regions. Furthermore, the ubiquitous expression of this subunit in the entire adult brain could explain the widespread and reproducible effects of indicaxanthin. On the other hand, the implication of an interplay between indicaxanthin with GABA system should be also considered. In this regard, an enhancement of GABA-currents induced by the flavone hispidulin, exerting anticonvulsive outcomes, was evidenced in an animal model of seizure (Kavvadias et al., 2004). Furthermore, the finding that flavonoids produced anxiolytic effect by virtue of an affinity for benzodiazepine site on GABA receptor (Medina et al., 1998) suggests a fine addressed action of PhC in modulating neuronal excitability. However, the possible interaction of indicaxanthin with other channels determining the excitability state of neurons cannot be ruled out and deserves further studies.

In the light of the present results, a noticeable direct neuromodulatory action for the indicaxanthin emerges. This influence on the cell excitability could have implications in some cellular dysfunctions induced by alterations of membrane mechanisms. Indeed, increased excitation could bring to excitotoxicity, especially in cortical areas, that triggers damaging and neurodegenerative disorders, such as Alzheimer disease, above all, but also epilepsy (Lipton and Rosenberg, 1994; Danysz and Parsons, 2003; Dong et al., 2009; Mehta et al., 2013). Since many beneficial neuroprotective effects of nutraceuticals are almost exclusively ascribed to their anti-inflammatory and antioxidant properties, the findings of a direct influence of indicaxanthin on the bioelectric activity of neurons shed new light on the possible mechanisms lending to dietary vegetables their favorable and healthy features. In particular, considering the prevalent inhibitory effect of indicaxanthin, the potential brake to excitatory transmission could constitute a further factor underpinning the benefits for maintenance of cell function. In this framework, inhibitory action of indicaxanthin in cortical and hippocampal areas, involved in cognitive functions and often affected by neurodegenerative deficits, represents an intriguing perspective for the use of this and, possibly, other phyto-nutrients in the implementation of anti-neurodegenerative strategies. Indeed, several researches already reported the positive effects of diets enriched with PhC on the brain function by improving telencephalic cognitive processes (Spencer, 2008). For example, in aged rats, the chronic supplementation with flavonoids from gingko biloba increased hippocampal LTP via the reduction of neuronal excitability (Williams et al., 2004). The altered excitability underlying age-related amyloid pathologies could also lead to epileptogenesis and some dietary plant-derived nutrients already revealed anticonvulsant effects (Kavvadias et al., 2004; Nassiri-Asl et al., 2008; Gurevicius et al., 2013; Diniz et al., 2015; Aggleton et al., 2016), also in aberrant cortico-thalamic hyperexcited connections (Gurevicius et al., 2013). Since this detrimental link between neurodegeneration and altered neuronal excitability could constitute one of the factors concurring to brain diseases, this study opens novel therapeutic perspectives and prompts future investigations about mechanism of action of indicaxanthin in the neuronal environment. Together with the neuroprotective action of nutraceuticals due to their antioxidant and anti-inflammatory properties, a further cellular target at synaptic level was individuated with a view to deepen knowledge on beneficial effects of phytonutrients on neuronal function.

## Author Contributions

GG, FC, MA and AA conducted the experiments. GG, FC, MA and PS performed the data analyses. FC, MA, PS, GF, LT and MAL designed the experiments. FC designed and directed the project. GG, FC and MA wrote the manuscript with input from all the authors. All authors read and approved the final manuscript.

## Conflict of Interest Statement

The authors declare that the research was conducted in the absence of any commercial or financial relationships that could be construed as a potential conflict of interest.
