# Supplementary figures and images for: Brain Distribution and Modulation of Neuronal Excitability by Indicaxanthin From Opuntia Ficus Indica Administered at Nutritionally-Relevant Amounts
Source: Front Aging Neurosci. 2018 May 9;10:133. doi: 10.3389/fnagi.2018.00133 (PMC5954040; doi:10.3389/fnagi.2018.00133)

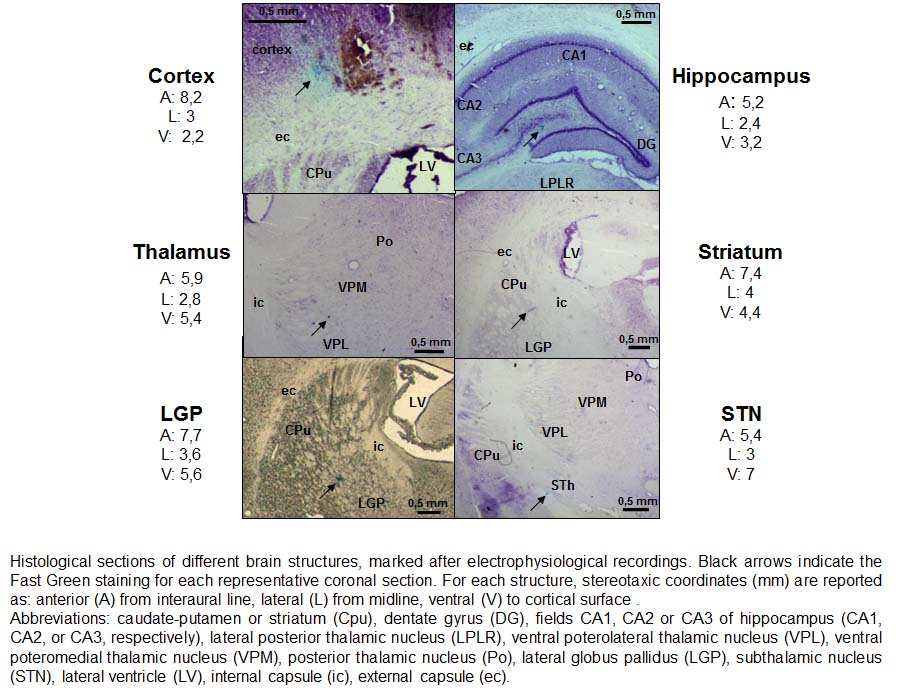

Supplement: Supplementary file 1 [file Image_1.JPEG]
